# pKa engineering of a near‐infrared fluorescent probe for sensitive imaging of oxidative stress in atherosclerotic plaques

**DOI:** 10.1002/smo2.70102

**Published:** 2026-09-07

**Authors:** Qianqian Yu, Jiahui Liang, Tijian Zhou, Qiaomeng Chen, Xuechun Yan, Zhikang Zheng, Huyou Wang, Jihong Liu, Yi Dong, Wei‐Hong Zhu, Qi Wang

**Affiliations:** ^1^ Key Laboratory for Advanced Materials and Joint International Research Laboratory of Precision Chemistry and Molecular Engineering Shanghai Key Laboratory of Functional Materials Chemistry Feringa Nobel Prize Scientist Joint Research Center Institute of Fine Chemicals Frontiers Science Center for Materiobiology and Dynamic Chemistry School of Chemistry and Molecular Engineering East China University of Science and Technology Shanghai China; ^2^ State Key Laboratory of Green Chemical Engineering and Industrial Catalysis Center of Photosensitive Chemicals Engineering East China University of Science and Technology Shanghai China; ^3^ Department of Ultrasound Xinhua Hospital Affiliated to Shanghai JiaoTong University School of Medicine Shanghai China

**Keywords:** atherosclerosis, near‐infrared fluorescent probe, pKa engineering, reactive oxygen species

## Abstract

Reactive oxygen species (ROS) are key mediators in the progression of atherosclerosis, making ROS imaging valuable for evaluating oxidative stress in plaques. However, most activatable fluorescent probes show limited fluorescence enhancement under physiological conditions because their fluorophores are insufficiently ionized at neutral pH, resulting in weak signal output and low sensitivity. To address this issue, we developed a pKa‐engineering strategy by introducing electron‐withdrawing fluorine atoms at the ortho‐positions of the phenolic hydroxyl group to construct the fluorophore DCM‐2F‐OH. The difluoro substitution lowered the pKa to 6.0, enabling complete ionization and strong near‐infrared fluorescence emission at physiological pH. Incorporation of a boronate ester trigger further afforded the activatable ROS probe DCM‐2F‐B. DCM‐2F‐B displayed a 12.8‐fold fluorescence turn‐on response toward ROS, significantly higher than that of the non‐fluorinated analog (2.5‐fold), along with a low detection limit (0.03 μM) and low cytotoxicity. The probe successfully visualized endogenous ROS in lipopolysaccharide (LPS)‐stimulated macrophages and detected elevated ROS levels in aortic valve tissues from apolipoprotein E knockout (ApoE^−/−^) mice, consistent with histopathological analysis. This work demonstrates that pKa engineering is an effective strategy for improving activatable fluorescent probes under physiological conditions and provides a useful tool for imaging oxidative stress in cardiovascular diseases.

## Graphical abstract

A pKa‐engineered near‐infrared fluorescent probe (DCM‐2F‐B) enables sensitive imaging of oxidative stress in atherosclerotic plaques. Difluoro substitution lowers the phenolic hydroxyl pKa from 9.1 to 6.0, achieving near‐complete ionization at physiological pH. The probe exhibited a 12.8‐fold fluorescence turn‐on response toward ROS, successfully detecting elevated ROS levels in both activated macrophages and aortic valve tissues of ApoE^−/−^ mice.

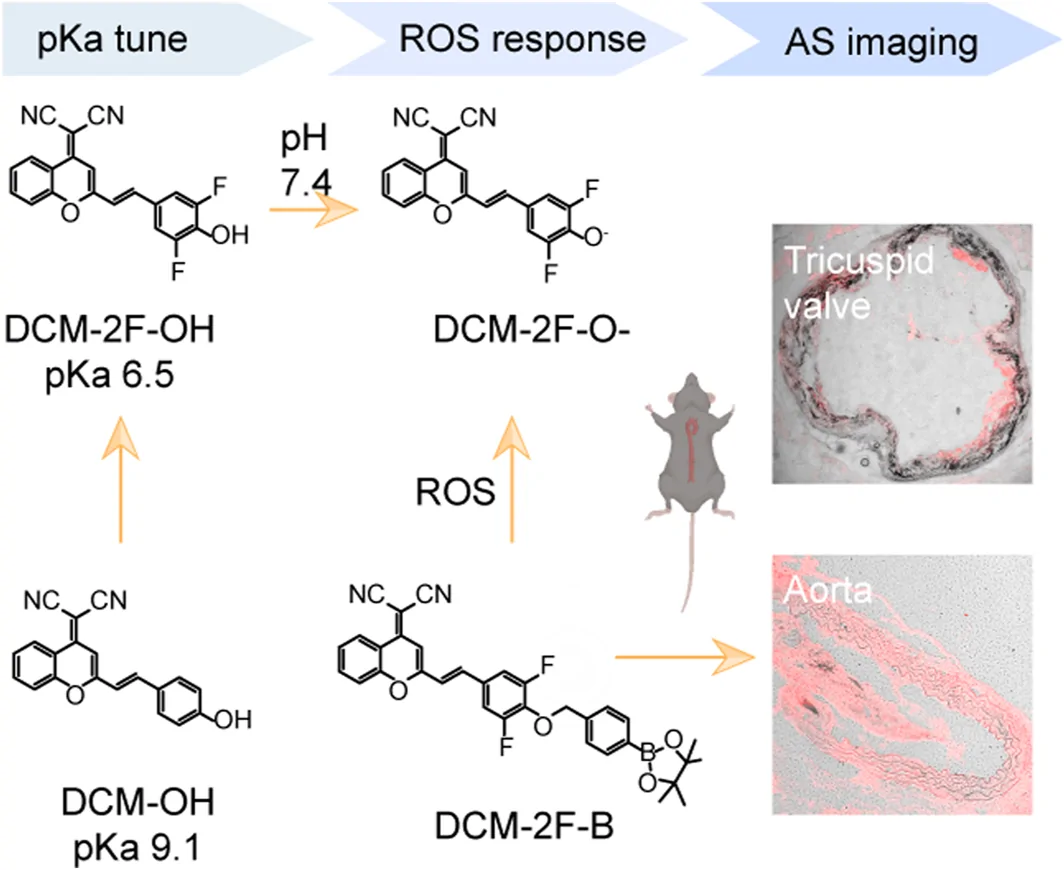

## 1 INTRODUCTION

Atherosclerosis (AS) is a major pathological basis of cardiovascular diseases such as myocardial infarction and stroke.[^1^, ^2^, ^3^] Plaque progression and instability are closely associated with oxidative stress in the inflammatory microenvironment, where activated macrophages produce excessive reactive oxygen species (ROS).[^4^, ^5^, ^6^] Elevated ROS levels not only damage vascular endothelial cells but also promote oxidized low‐density lipoprotein formation and plaque rupture.[^7^, ^8^, ^9^, ^10^] Therefore, direct visualization of ROS within AS plaques is of great significance for early diagnosis and risk assessment.[^11^, ^12^, ^13^] Near‐infrared (NIR) fluorescence imaging has attracted considerable attention for biomarker detection because of its low tissue autofluorescence, relatively deep tissue penetration, and real‐time imaging capability.[^14^, ^15^, ^16^] However, the development of highly sensitive ROS‐activatable NIR probes under physiological conditions remains a significant challenge.[^17^, ^18^, ^19^]

To address ROS imaging, a variety of activatable fluorescent probes based on intramolecular charge transfer (ICT) fluorophores have been developed.[^20^, ^21^, ^22^, ^23^, ^24^] Among them, dicyanomethylene‐4H‐pyran (DCM)‐based fluorophores are particularly attractive due to their facile synthesis, favorable photostability, and large Stokes shifts.[^25^, ^26^, ^27^] Nevertheless, the practical application of conventional DCM‐OH fluorophores is hindered by their high pKa value (approximately 9.1).[^28^, ^29^, ^30^] Since the physiological environment typically maintains a pH of 7.4, these fluorophores remain largely in their protonated, non‐fluorescent state, resulting in weak fluorescence output even after the ROS‐responsive trigger is cleaved.[^31^, ^32^] This insufficient ionization at physiological pH severely limits the fluorescence “turn‐on” efficiency and imaging sensitivity, rendering current probes inadequate for the precise detection of oxidative stress in AS lesions.

**FIGURE 1:**
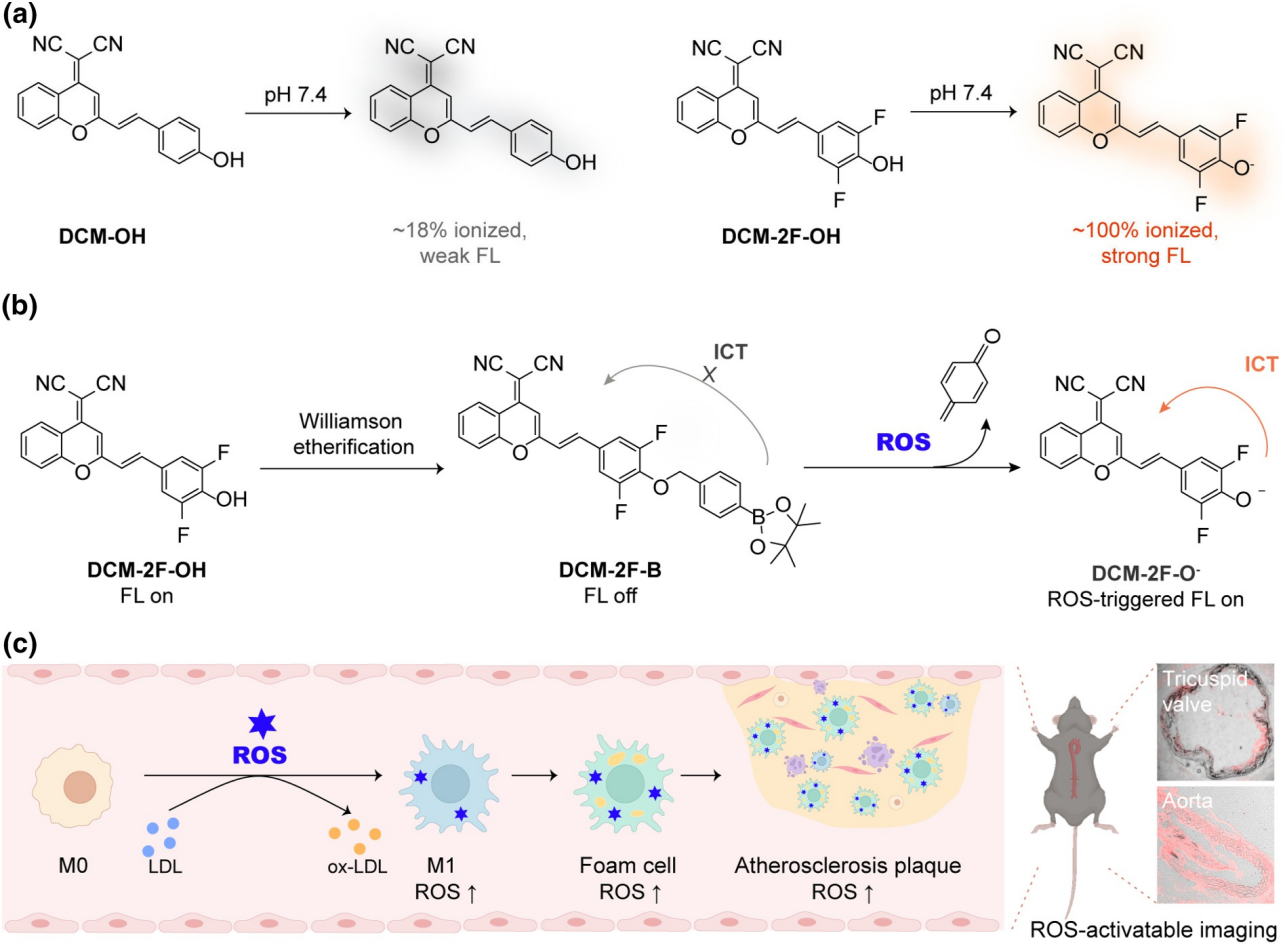
pKa‐tuned ROS‐activatable NIR probe DCM‐2F‐B for atherosclerotic plaque imaging. (a) Molecular design strategy. Conventional DCM‐OH has a high pKa (9.1) and remains weakly fluorescent at pH 7.4. Fluorine substitution at ortho‐positions lowers pKa to 6.0, enabling nearly complete ionization and strong NIR fluorescence. (b) ROS‐responsive mechanism. The probe DCM‐2F‐B was prepared via Williamson etherification. The boronate ester locks the probe in a fluorescence‐off state by blocking ICT. ROS trigger oxidative cleavage, releasing DCM‐2F‐OH and restoring ICT, resulting in fluorescence turn‐on. (c) Imaging applications. DCM‐2F‐B successfully images endogenous ROS in LPS‐activated macrophages (cellular level) and detects elevated ROS levels in both aortic and tricuspid valve tissues of atherosclerosis mice. DCM, dicyanomethylene‐4H‐pyran; ICT, intramolecular charge transfer; NIR, near‐infrared; ROS, reactive oxygen species.

To overcome this limitation, we employed a pKa‐engineering strategy by introducing electron‐withdrawing fluorine atoms at the ortho‐positions of the phenolic hydroxyl group to construct the low‐pKa fluorophore DCM‐2F‐OH (Figure 1a). The difluoro substitution reduced the pKa to 6.0, enabling nearly complete ionization and enhanced fluorescence emission at physiological pH. Based on this optimized fluorophore, a boronate ester moiety was further incorporated as a ROS‐responsive trigger to develop the activatable NIR probe DCM‐2F‐B (Figure 1b). Compared with its non‐fluorinated counterpart, DCM‐2F‐B exhibited a markedly enhanced fluorescence response to ROS, enabling the high‐contrast visualization of endogenous ROS in activated macrophages and pathological ROS accumulation in the aortic valves of ApoE^−/−^ mice (Figure 1c). This work demonstrates that pKa engineering is an effective strategy for improving activatable fluorescent probes under physiological conditions and provides a promising tool for imaging oxidative stress in cardiovascular diseases.

## 2 RESULTS AND DISCUSSION

### 2.1 pKa‐engineered ROS sensing mechanism and performance

**FIGURE 2:**
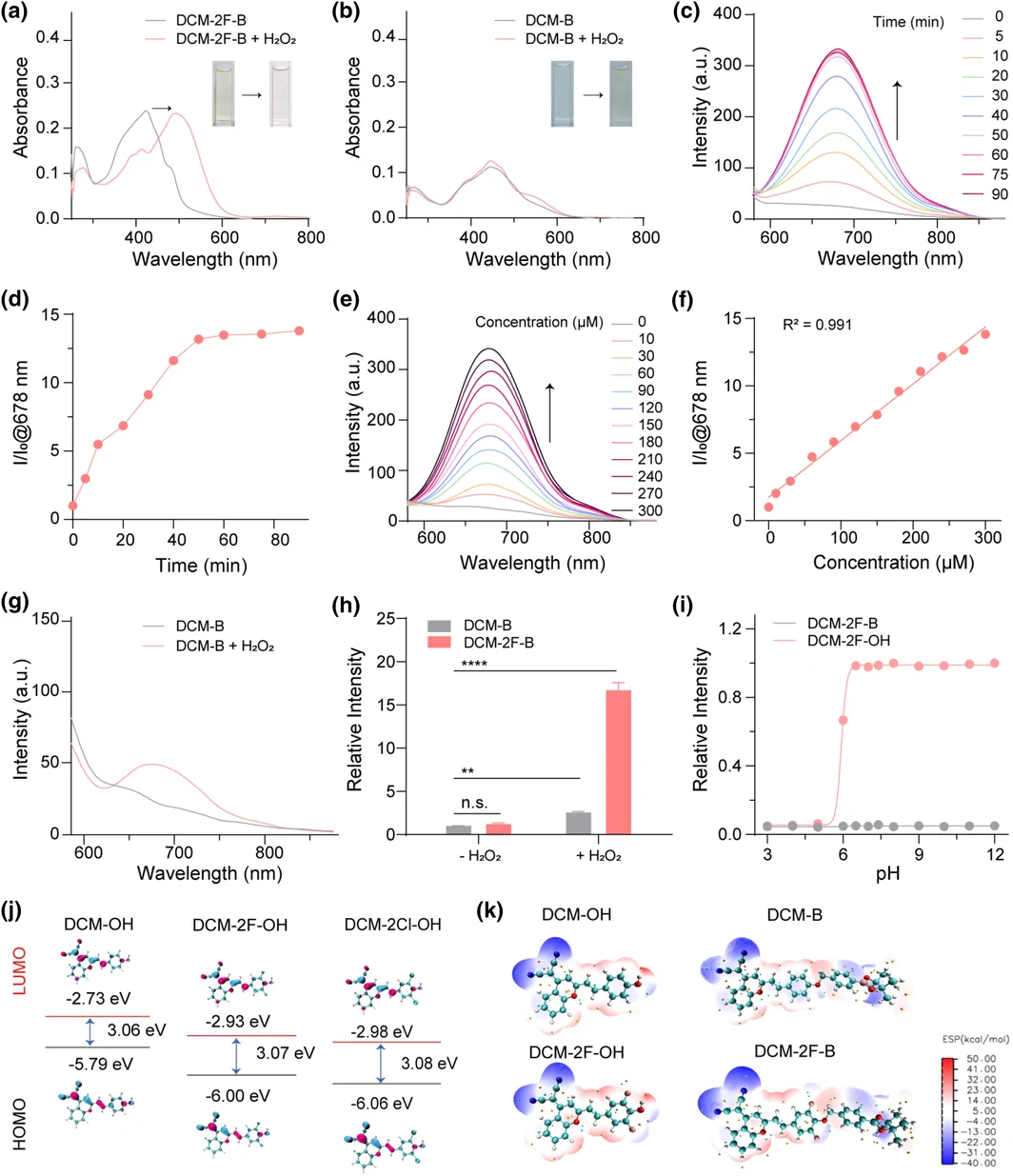
Spectroscopic response and sensing performance of DCM‐2F‐B and DCM‐B. (a) Absorption spectra of DCM‐2F‐B before and after H_2_O_2_ treatment. Inset: photographs of DCM‐2F‐B solutions before and after H_2_O_2_ addition (300 μM, 60 min). (b) Absorption spectra of DCM‐B under the same conditions. (c) Time dependent fluorescence intensity of DCM‐2F‐B at 678 nm upon H_2_O_2_ addition. (d) Kinetic profile of the fluorescence turn‐on response. (e) Fluorescence spectra of DCM‐2F‐B with increasing H_2_O_2_ concentration (0–300 μM). (f) Linear relationship between fluorescence intensity and H_2_O_2_ concentration (*R* ^2^ = 0.991). Detection limit: 0.03 μM. (g) Fluorescence spectra of DCM‐B before and after H_2_O_2_ incubation (60 min, 300 μM). (h) Comparison of fluorescence turn‐on ratios. (i) pH dependent fluorescence intensity. (j) DFT‐calculated frontier molecular orbital energy levels of DCM‐OH, DCM‐2F‐OH, and DCM‐2Cl‐OH. (k) Molecular electrostatic potential maps of DCM‐OH, DCM‐2F‐OH, and their boronate ester derivatives. Fluorescence responses of DCM‐2F‐B were measured at *λ*_ex_ = 505 nm and *λ*_em_ = 678 nm, while DCM‐B was measured at *λ*_ex_ = 532 nm and *λ*_em_ = 683 nm. All quantitative data are presented as mean ± SD from three independent experiments. Statistical significance for the turn‐on ratio comparison was evaluated using a two‐tailed Student's *t*‐test. Significance levels are denoted as **p < 0.01 and ****p < 0.0001, while n.s. indicates no statistically significant difference. DCM, dicyanomethylene‐4H‐pyran.

DCM‐2F‐B and the non‐fluorinated control probe DCM‐B were synthesized via Knoevenagel condensation followed by nucleophilic substitution with 4‐(bromomethyl)phenylboronic acid pinacol ester (Scheme S1, Figures S1–S11).[^33^, ^34^, ^35^] The ROS‐responsive spectroscopic behavior of DCM‐2F‐B was first investigated using H_2_O_2_ as a model analyte. As shown in Figure 2a, DCM‐2F‐B exhibited a maximum absorption peak at 422 nm, indicating that the boronate ester‐protected phenolic hydroxyl group effectively suppressed the ICT process. Upon incubation with H_2_O_2_ (300 μM) at 37°C for 60 min, the absorption maximum markedly red‐shifted to 490 nm, accompanied by a distinct color change from pale yellow to pink (inset in Figure 2a). This spectral transition is attributed to the oxidative cleavage of the boronate ester group, which releases DCM‐2F‐OH and restores the donor−π−acceptor (D−π−A) structure. In contrast, the non‐fluorinated control probe DCM‐B showed almost no absorption shift after H_2_O_2_ treatment (Figure 2b), suggesting that fluorine substitution plays a critical role in restoring the ICT process under physiological conditions.

The fluorescence sensing performance of DCM‐2F‐B was subsequently evaluated. As shown in Figure 2c,d, the probe initially exhibited negligible fluorescence, whereas rapid fluorescence enhancement was observed after H_2_O_2_ addition and reached a plateau within 60 min. Therefore, 60 min was selected as the optimal incubation time for subsequent experiments. Upon increasing the H_2_O_2_ concentration from 0 to 300 μM, the fluorescence intensity at 678 nm gradually increased (Figure 2e) and displayed a good linear relationship with H_2_O_2_ concentration (*R* ^2^ = 0.991, Figure 2f). The detection limit was calculated to be 0.03 μM using the 3*σ*/slope method, indicating high sensitivity toward H_2_O_2_ detection. Notably, DCM‐2F‐B exhibited an approximately 12.8‐fold fluorescence turn‐on response upon H_2_O_2_ activation, whereas the non‐fluorinated probe DCM‐B showed only a 2.3‐fold enhancement under identical conditions (Figure 2g,h), demonstrating the substantial improvement achieved through fluorine substitution.

To elucidate the origin of this enhanced fluorescence responsiveness, the pH‐dependent fluorescence behaviors of the corresponding fluorophores were investigated. DCM‐2F‐OH displayed a typical sigmoidal fluorescence‐pH profile with a pKa value of 6.0, which is markedly lower than that of DCM‐OH (pKa = 9.1) (Figure 2i). The reduced pKa enables nearly complete ionization of DCM‐2F‐OH at physiological pH, thereby generating the strongly electron‐donating phenolate form and efficiently activating the ICT process. In contrast, DCM‐OH remains predominantly in its weak fluorescent‐neutral form under the same conditions. Moreover, DCM‐2F‐B maintained consistently low fluorescence over a broad pH range, confirming that the protected probe remained in a stable fluorescence‐off state prior to activation. For comparison, DCM‐2Cl‐OH exhibited an intermediate pKa value of approximately 6.5 (Figure S12), consistent with the electronic effect of chlorine substitution.

To further investigate the electronic effects of halogen substitution, density functional theory calculations were conducted for both the free fluorophores (DCM‐OH, DCM‐2F‐OH, and DCM‐2Cl‐OH) and their corresponding boronate ester‐masked probes (DCM‐B, DCM‐2F‐B, and DCM‐2Cl‐B). As shown in Figures 2j and S13, halogen substitution consistently lowered both the highest occupied molecular orbital and lowest unoccupied molecular orbital energy levels across both series, which is in good agreement with the experimentally observed spectral shifts. Molecular electrostatic potential analysis further revealed increased electron density around the phenolic oxygen atom after fluorine substitution (Figures 2k and S14), which favors stabilization of the phenolate anion and facilitates deprotonation. In contrast, introduction of the boronate ester group significantly reduced electron density around the oxygen atom, thereby suppressing the ICT process and maintaining the probe in a fluorescence‐off state before activation. These theoretical results are consistent with the spectroscopic experiments and further support that fluorine substitution enhances ROS responsiveness by lowering the fluorophore pKa and promoting ionization under physiological conditions.

### 2.2 Response specificity and biological stability

**FIGURE 3:**
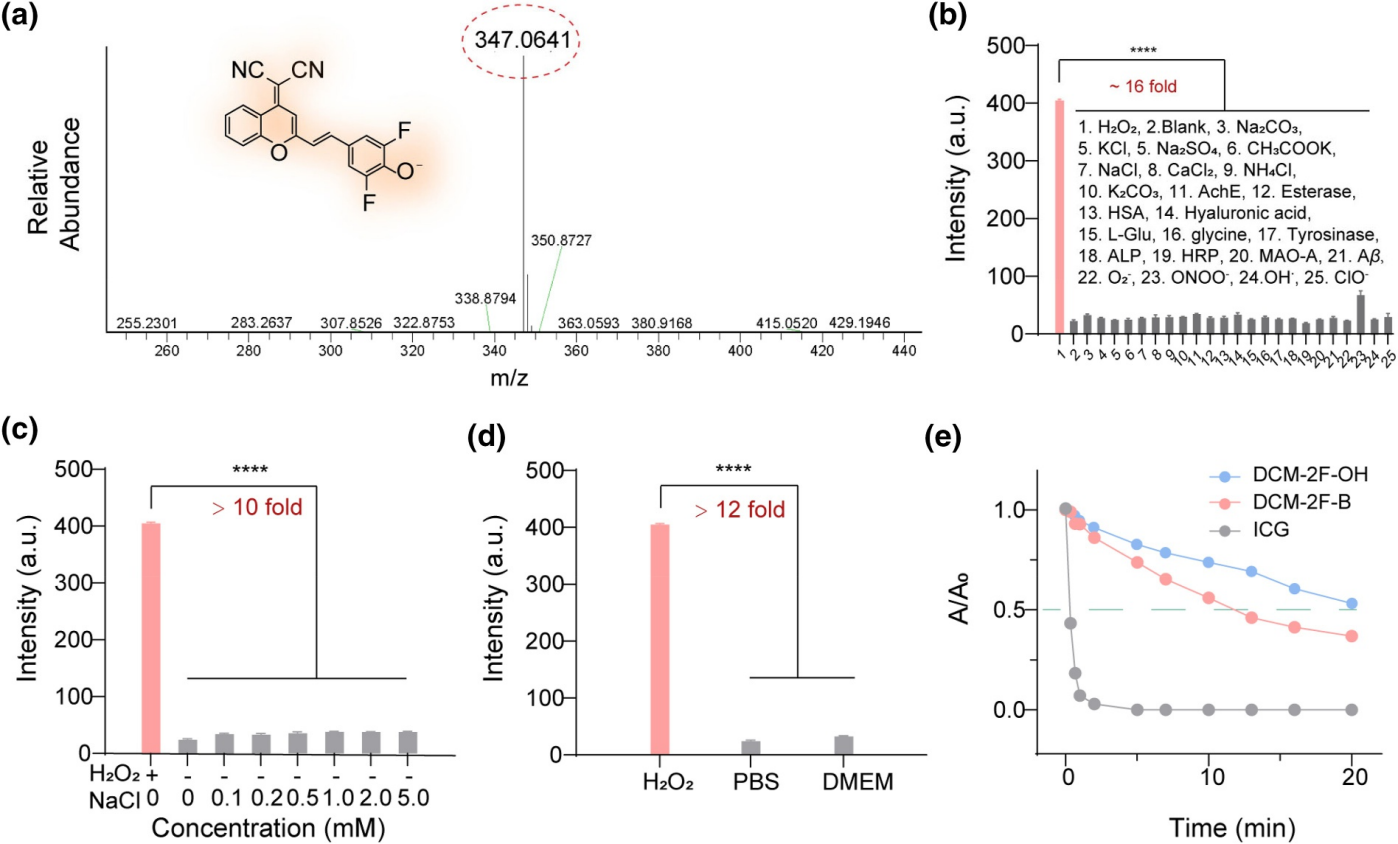
Response mechanism, selectivity and stability of DCM‐2F‐B. (a) High resolution mass spectrum confirming the release of DCM‐2F‐OH upon H_2_O_2_ stimulation. (b) Fluorescence selectivity toward H_2_O_2_ over various interfering substances. (c) Fluorescence stability of DCM‐2F‐B under varying concentrations of NaCl (0–5.0 mM). (d) Fluorescence stability in cell culture medium containing serum. (e) Photostability comparison of DCM‐2F‐B, DCM‐2F‐OH, and indocyanine green under continuous illumination. Fluorescence responses of DCM‐2F‐B were measured at *λ*_ex_ = 505 nm and *λ*_em_ = 678 nm. Fluorescence intensities for each interferent represent mean ± SD from three independent experiments; significance for the selectivity comparison was determined by one‐way ANOVA, with significance defined as ****p < 0.0001. ANOVA, analysis of variance; DCM, dicyanomethylene‐4H‐pyran.

To verify the activation mechanism of DCM‐2F‐B, high‐resolution mass spectrometry was performed before and after the reaction with H_2_O_2_. As shown in Figure 3a, incubation of DCM‐2F‐B with H_2_O_2_ generated a prominent peak at m/z = 347.0641 in the negative ion mode, which agrees well with the calculated mass of [DCM‐2F‐OH–H]^−^ (m/z = 347.0627). This result confirms that H_2_O_2_ specifically triggers oxidative cleavage of the boronate ester group to release DCM‐2F‐OH, thereby restoring the fluorescent ICT process.

The response selectivity of DCM‐2F‐B toward H_2_O_2_ was subsequently evaluated against various biologically relevant interferents, including inorganic salts, enzymes, amino acids, and disease‐related biomolecules. As shown in Figure 3b, only H_2_O_2_ induced a significant fluorescence enhancement (∼16‐fold), whereas all other tested substances caused negligible signal changes. These results demonstrate that DCM‐2F‐B possesses high selectivity for H_2_O_2_ based on the specific oxidative hydrolysis of the boronate ester recognition unit.

The stability and biological applicability of DCM‐2F‐B were further investigated under physiological conditions. As shown in Figure 3c, the probe maintained excellent stability over a broad pH range and under different salt concentrations, while consistently remaining in a low‐fluorescence state before activation. Moreover, no noticeable fluorescence change was observed after incubation in Dulbecco's modified Eagle medium containing 10% fetal bovine serum for 12 h (Figure 3d), confirming good stability in complex biological media. The photostability of DCM‐2F‐B was also evaluated using indocyanine green (ICG) as a reference dye. Under continuous irradiation, DCM‐2F‐B exhibited markedly slower photobleaching than ICG and retained approximately 37% of its initial absorbance after 20 min of illumination (Figure 3e). The photobleaching half‐life of DCM‐2F‐B was estimated to be approximately 12 min, and that of its hydrolysis product DCM‐2F‐OH was approximately 20 min, which is substantially longer than that of ICG (∼0.5 min). These results demonstrate that DCM‐2F‐B possesses excellent selectivity and stability for long‐term biological imaging applications.

### 2.3 In vitro imaging and quantitative detection of endogenous ROS in living cells

**FIGURE 4:**
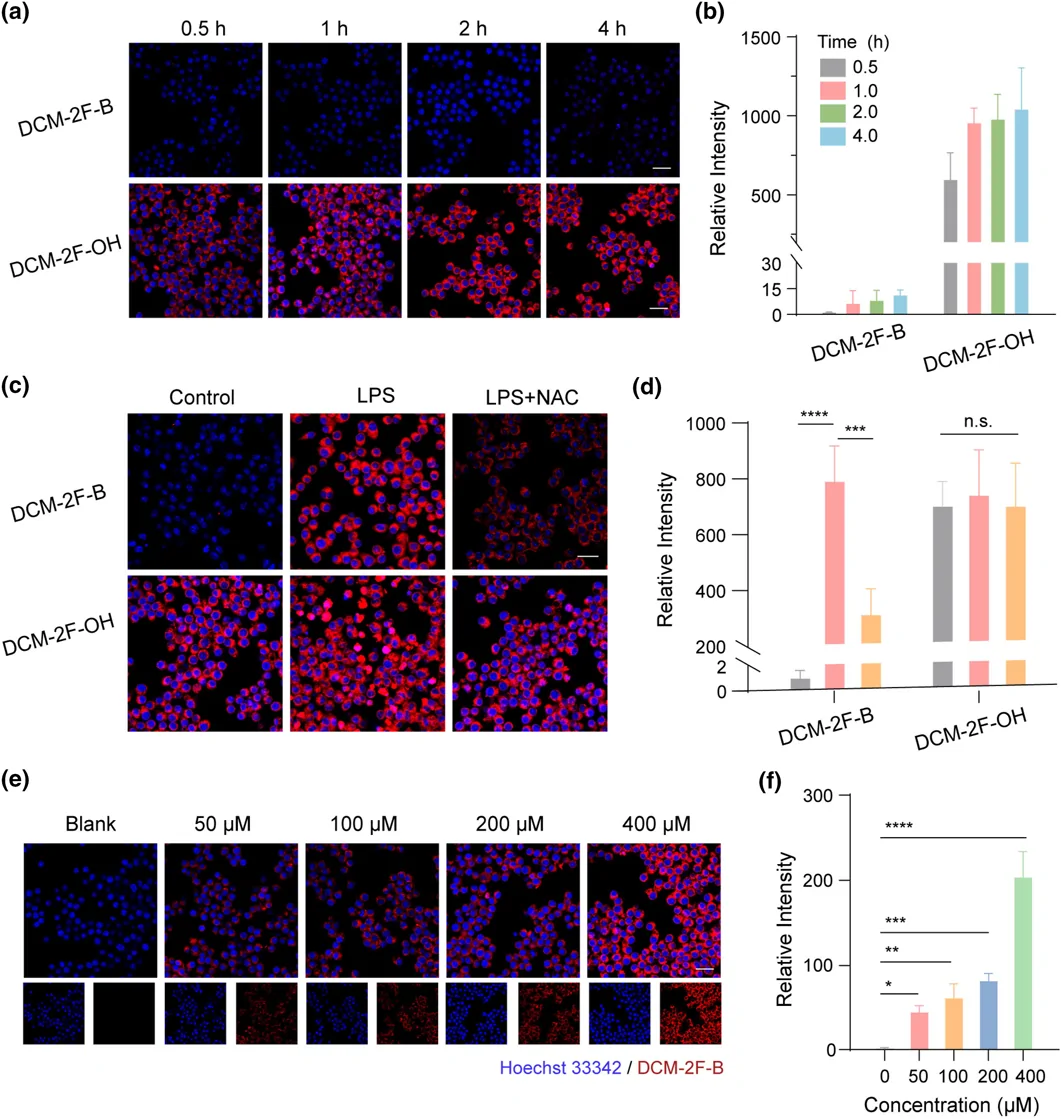
In vitro fluorescence imaging of ROS using DCM‐2F‐B. (a) Uptake kinetics of DCM‐2F‐B and DCM‐2F‐OH in RAW264.7 macrophages (0.5–4 h). (b) Quantitative comparison of fluorescence intensity between DCM‐2F‐B and DCM‐2F‐OH. (c) CLSM images of LPS induced endogenous ROS response with or without N‐acetylcysteine pretreatment. (d) Quantitative analysis of relative fluorescence intensity from panel (c). (e) CLSM images of cells stimulated with varying concentrations of H_2_O_2_ (0–400 μM). (f) Quantitative analysis of fluorescence enhancement at different H_2_O_2_ concentrations. Scale bars: 50 μm. The data are expressed as the mean ± SD. Statistical significance was determined using Student's *t*‐test for pairwise comparisons, and a one‐way ANOVA for multiple groups. *p < 0.05, **p < 0.01, ***p < 0.001, and ****p < 0.0001. Concordant results were obtained from three independent experiments. ANOVA, analysis of variance; CLSM, confocal laser scanning microscopy; DCM, dicyanomethylene‐4H‐pyran; ROS, reactive oxygen species.

RAW264.7 macrophages were first used to evaluate the cellular uptake, biocompatibility, and imaging behavior of DCM‐2F‐B. As shown in Figure 4a, fluorescence intensity in the probe‐treated group gradually increased within 0.5 h and then reached a plateau, indicating efficient cellular uptake and activation. Therefore, an incubation time of 1 h was selected for subsequent experiments. In contrast, cells treated with DCM‐2F‐OH showed strong fluorescence signals within 0.5 h, approximately 600‐fold higher than those of the DCM‐2F‐B treated group (Figure 4b), confirming the dark‐state nature of the probe before activation. The biocompatibility of DCM‐2F‐B was then evaluated using 3‐(4,5‐dimethylthiazol‐2‐yl)‐2,5‐diphenyltetrazolium bromide assays. As shown in Figure S15, cell viability remained above 93% at 10 μM and exceeded 85% even at 20 μM for all tested derivatives, indicating negligible cytotoxicity within the working concentration range.

The ability of DCM‐2F‐B to image endogenous ROS was first investigated in lipopolysaccharide (LPS)‐stimulated macrophages. As shown in Figure 4c,d, negligible fluorescence was observed in the control group, whereas a strong fluorescence signal was detected after LPS stimulation, indicating effective activation by intracellular ROS. To verify specificity, cells were pretreated with the ROS scavenger N‐acetylcysteine (NAC), which significantly suppressed fluorescence intensity, confirming that the signal originates from ROS‐responsive activation.

To further validate the robustness and generality of the probe response, additional experiments were performed under different stimulation conditions and incubation times. As shown in Figure S16, even with a shorter probe incubation time of 30 min, strong fluorescence activation was still observed upon exogenous H_2_O_2_ stimulation (approximately 24‐fold enhancement). Moreover, phorbol 12‐myristate 13‐acetate‐induced endogenous ROS also triggered a clear fluorescence response (approximately 12‐fold increase). Importantly, ROS scavenging and inhibition experiments further confirmed the specificity: NAC completely suppressed fluorescence to baseline levels, while the nicotinamide adenine dinucleotide phosphate oxidase inhibitor apocynin significantly reduced the signal to approximately 4‐fold. These results are consistent with those obtained under 1 h incubation conditions, demonstrating the robustness and reliability of DCM‐2F‐B for ROS imaging under different biological stimulation models.

Finally, the quantitative imaging capability of DCM‐2F‐B was evaluated using exogenous H_2_O_2_ stimulation (Figure 4e,f). A clear concentration‐dependent fluorescence increase was observed, with a linear response in the range of 0–200 μM (*R* ^2^ = 0.97) and a detection limit of 6.5 μM, confirming its ability for quantitative ROS analysis in living cells.

### 2.4 Ex vivo imaging of ROS in atherosclerotic plaque tissues

**FIGURE 5:**
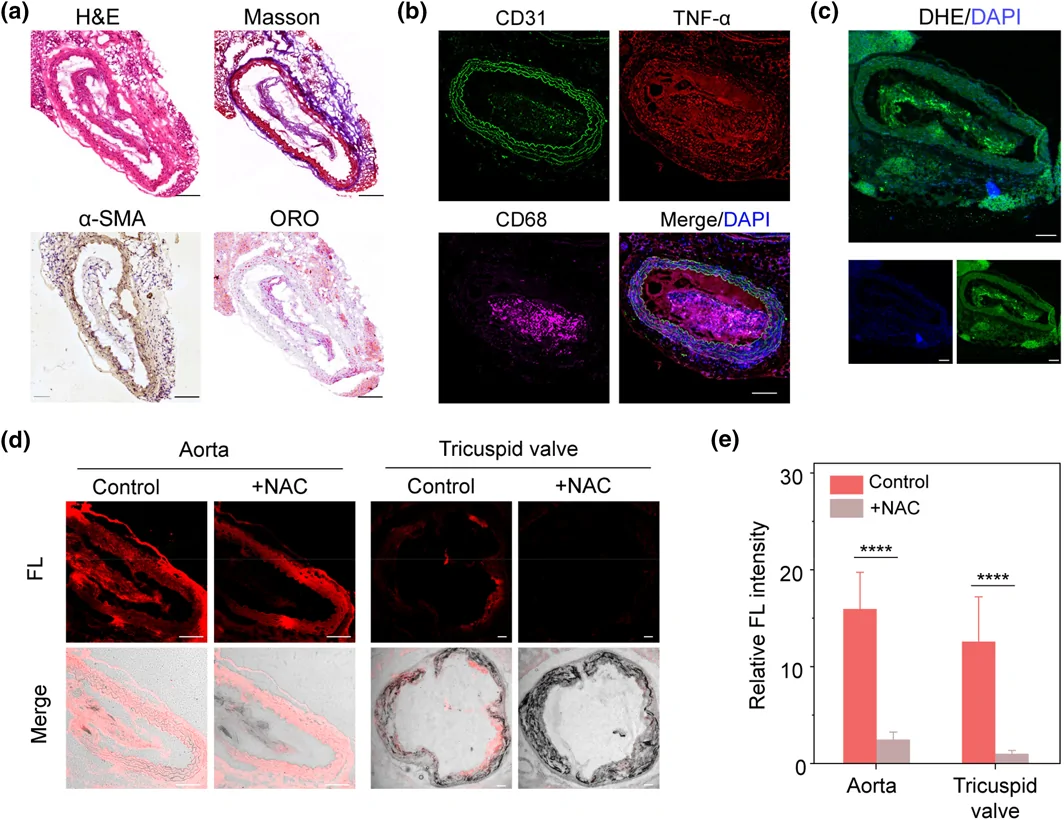
Histopathological characterization and ROS imaging of atherosclerosis plaque tissues. (a) H&E, Masson, *α*‐SMA, and Oil Red O staining of aortic valve tissues. Scale bar: 50 μm. (b) CD31, TNF‐*α*, and CD68 immunofluorescence staining. Scale bar: 50 μm. (c) Dihydroethidium staining showing elevated ROS levels in aortic valve tissues. Scale bar: 50 μm. (d) Fluorescence imaging of DCM‐2F‐B in aortic valve tissues with and without N‐acetylcysteine pretreatment. Scale bar: 100 μm. (e) Quantitative analysis of relative fluorescence intensity. The data are expressed as the mean ± SD. Statistical significance was determined using Student's *t*‐test for pairwise comparisons, and a one‐way ANOVA for multiple groups. ****p < 0.0001. Three mice in each group (*n* = 3). ANOVA, analysis of variance; DCM, dicyanomethylene‐4H‐pyran; ROS, reactive oxygen species.

To evaluate the capability of DCM‐2F‐B for detecting ROS in pathological environment, ApoE^−/−^ mouse model was employed. As recently summarized, rational design and performance validation of activatable fluorescent probes in pathological tissues represent a core strategy to achieve reliable biosensing in complex biological microenvironments.^[36]^ Histological analyses confirmed the successful establishment of the disease model. As shown in Figure 5a, H&E staining revealed significant inflammatory cell infiltration, while Masson's trichrome and Oil Red O staining indicated marked collagen deposition and lipid accumulation, respectively. In addition, *α*‐SMA staining showed reduced smooth muscle cell content, suggesting vascular wall remodeling and plaque instability. These results collectively confirm the presence of advanced atherosclerotic lesions.

To further verify the inflammatory and oxidative microenvironments, immunofluorescence and ROS‐specific staining were performed. As shown in Figure 5b, strong signals of TNF‐*α* and CD68 indicated pronounced inflammation and macrophage infiltration within the valve tissues. Meanwhile, dihydroethidium staining revealed significantly elevated ROS levels in diseased tissues compared with controls, confirming a highly oxidative microenvironment characteristic of atherosclerotic lesions (Figure 5c).

Having confirmed the pathological and oxidative features of the model, the performance of DCM‐2F‐B was next evaluated in ex vivo tissues. As shown in Figure 5d, a strong fluorescence signal was observed in aortic valve tissues incubated with DCM‐2F‐B, with approximately 16‐fold enhancement compared with baseline. A similarly elevated response (∼13‐fold) was detected in the tricuspid valve tissues, demonstrating that the probe can effectively visualize ROS in different valve regions of atherosclerotic mice.

To validate the specificity of the fluorescence response, NAC pretreatment was performed prior to tissue collection. As shown in Figure 5e, NAC treatment dramatically suppressed fluorescence signals in both aortic and tricuspid valve tissues, reducing them to near‐baseline levels (****p < 0.0001). These results confirm that the fluorescence activation of DCM‐2F‐B in atherosclerotic tissues is specifically driven by elevated ROS levels. This explicit ROS‐responsive performance in ex vivo atherosclerotic lesions further extends the category of activatable fluorescent probes for disease‐associated biomarker imaging in pathological tissues, which is consistent with the recently reported strategy of engineering target‐activatable NIR probes for visualized tracking of disease‐related biological events in intact tissues.^[37]^

## 3 CONCLUSION

In conclusion, we developed a pKa‐engineered activatable fluorescent probe, DCM‐2F‐B, for the sensitive imaging of ROS in atherosclerotic plaques. By introducing electron‐withdrawing fluorine atoms at the ortho‐position of the phenolic hydroxyl group, the fluorophore pKa was effectively reduced, enabling efficient ionization and strong fluorescence output under physiological conditions. The probe exhibited a 12.8‐fold fluorescence turn‐on response toward H_2_O_2_ with a detection limit of 0.03 μM. In an AS mouse model, DCM‐2F‐B enabled clear visualization of elevated ROS levels in aortic valve tissues, showing approximately 16‐fold fluorescence enhancement compared with control tissues, in agreement with histopathological and ROS‐specific staining analyses. These results demonstrate its reliable performance for ROS imaging in complex pathological environments. Overall, this work provides a robust molecular tool for ROS imaging in cardiovascular disease and highlights pKa engineering as a general strategy for optimizing activatable fluorescent probes. The pKa‐engineering strategy may facilitate the development of advanced probes for deep‐tissue and disease‐targeted imaging applications.

## CONFLICT OF INTEREST STATEMENT

The authors declare no conflicts of interest.

## ETHICS STATEMENT

All animal experiments were approved by the Animal Care and Use Committee of Shanghai Cancer Institute (SYXK(Shanghai)2017‐0011) and performed in accordance with the relevant guidelines. No human subjects were involved in this study.

## Supporting information

Supporting Information S1

## Data Availability

The data that support the findings of this study are available in Supporting Information S1 of this article.
