# Supplementary material for: pKa engineering of a near‐infrared fluorescent probe for sensitive imaging of oxidative stress in atherosclerotic plaques
Source: Smart Mol. 2026 Sep 7:e70102. Online ahead of print. doi: 10.1002/smo2.70102 (PMC13550858; doi:10.1002/smo2.70102)
Supplement: Supplementary file 1 [file SMO2-9999-0-s001.docx]

pKa-Engineering of a Near-Infrared Fluorescent Probe for Sensitive Imaging of Oxidative Stress in Atherosclerotic Plaques

Qianqian Yu^a^, Jiahui Liang^a^, Tijian Zhou^a^, Qiaomeng Chen^a^, Xuechun Yan^a^, Zhikang Zheng^a^, Huyou Wang^a^, Jihong Liu* ^a, b^, Yi Dong* ^c^, Wei-Hong Zhu^a, b^, and Qi Wang* ^a, b^

Q. Yu, J. Liang, T. Zhou, Q. Chen, X. Yan, Z. Zheng, H. Wang, J. Liu, W.-H. Zhu, and Q. Wang

^a^ Key Laboratory for Advanced Materials and Joint International Research Laboratory of Precision Chemistry and Molecular Engineering, Shanghai Key Laboratory of Functional Materials Chemistry, Feringa Nobel Prize Scientist Joint Research Center, Institute of Fine Chemicals, Frontiers Science Center for Materiobiology and Dynamic Chemistry, School of Chemistry and Molecular Engineering, East China University of Science and Technology, Shanghai 200237, China

J. Liu, W.-H. Zhu, and Q. Wang

^b^ State Key Laboratory of Green Chemical Engineering and Industrial Catalysis, Center of Photosensitive Chemicals Engineering, East China University of Science and Technology, Shanghai 200237, China

Y. Dong

^c^ Department of Ultrasound, Xinhua Hospital Affiliated to Shanghai JiaoTong University School of Medicine, 1665th Kongjiang Road, Shanghai 200092, China.

Email: jihong0829@ecust.edu.cn, dong.yi@shsmu.edu.cn, wangqi@ecust.edu.cn

**1.** **Synthesis of** **DCM-2F-B and DCM-B**

**Scheme S1.** Synthetic route of DCM-2F-B and DCM-B.

**Synthesis of Probe DCM-B**

DCM (40.0 mg, 0.192 mmol) and 4-hydroxybenzaldehyde (35.2 mg, 0.288 mmol) were added to a 25 mL Schlenk tube and dissolved in anhydrous ethanol (7 mL). Piperidine (0.5 mL) was added dropwise, and the mixture was stirred under reflux for 12 h. After completion of the reaction, the solvent was removed under reduced pressure. The residue was dissolved in dichloromethane (30 mL), adsorbed onto silica gel, and purified by column chromatography (DCM/MeOH, v/v, 20/1) to afford the product as a yellow solid (27.6 mg, yield 43.5%). ^1^H NMR (400 MHz, DMSO-*d*_6_, ppm): *δ* 10.16 (s, 1H, -OH), 8.70 (d, *J* = 7.2 Hz, 1H, phenyl-H), 7.91-7.87 (m, 2H, phenyl-H), 7.77-7.75 (m, 1H, phenyl-H), 7.68-7.56 (m, 4H, phenyl-H and alkene-H), 7.22 (d, *J* = 16.0 Hz, 1H, alkene-H), 6.92 (s, 1H, pyran-H), 6.85 (d, *J* = 8.4 Hz, 2H, phenyl-H). ^13^C NMR (400 MHz, DMSO-*d*_6_, ppm): *δ* 160.51, 159.33, 153.29, 152.47, 139.72, 135.72, 130.82, 126.57, 126.51, 125.05, 119.46, 117.85, 117.57, 116.49, 116.36, 106.16, 59.56.

DCM-OH (20.0 mg, 0.0640 mmol), 4-(bromomethyl)phenylboronic acid pinacol ester (28.5 mg, 0.0960 mmol), anhydrous sodium sulfate (22.7 mg, 0.160 mmol), and cesium carbonate (104.3 mg, 0.320 mmol) were added to a 25 mL two-necked flask, and dissolved in acetonitrile (20 mL). The mixture was stirred at room temperature for 24 h. The reaction mixture was filtered to remove inorganic salts, and the filter cake was washed with dichloromethane. The combined organic phases were concentrated and purified by column chromatography (DCM/MeOH, v/v, 50/1) to afford the product as a yellow solid (9.9 mg, yield 29.4%). ^1^H NMR (400 MHz, CDCl_3_, ppm): *δ* 8.91 (d, *J* = 8.0 Hz, 1H, phenyl-H), 7.84 (d, *J* = 7.6 Hz, 2H, phenyl-H), 7.71 (t, *J* = 7.6 Hz, 1H, phenyl-H), 7.52 (m, 4H, alkene-H, phenyl-H), 7.43 (t, *J* = 8.0 Hz, 3H, phenyl-H), 7.01 (d, *J* = 8.8 Hz, 2H, phenyl-H), 6.83 (s, 1H, pyran-H), 6.66 (d, *J* = 16.0 Hz, 1H, alkene-H), 5.16 (s, 2H, -CH_2_-), 1.35 (s, 12H, -Bpin). ^13^C NMR (400 MHz, CDCl_3_, ppm): *δ* 160.77, 157.94, 152.92, 152.36, 139.43, 138.69, 135.14, 134.53, 129.71, 127.65, 126.50, 125.89, 125.84, 118.57, 117.90, 116.99, 116.41, 115.91, 115.59, 106.29, 83.91, 70.05, 62.07, 24.88. HRMS (ESI positive ion mode for [M+H]⁺): Calcd. for C_33_H_30_N_2_O_4_B^+^: 529.2293; found: 529.2297.

**Synthesis of Probe DCM-2F-B**

DCM (50.0 mg, 0.240 mmol) and 3,5-difluoro-4-hydroxybenzaldehyde (56.9 mg, 0.360 mmol) were added to a 25 mL Schlenk tube and dissolved in anhydrous ethanol (7 mL). Piperidine (0.5 mL) was added dropwise, and the mixture was heated under reflux for 12 h. After completion of the reaction, the solvent was removed under reduced pressure. The residue was purified by column chromatography (DCM/MeOH, v/v, 20/1) to afford the product DCM-2F-OH as a yellow solid (26.6 mg, yield 31.8%). ^1^H NMR (400 MHz, DMSO-*d*_6_, ppm): *δ* 10.90 (s, 1H, -OH), 8.70 (d, *J* = 8.4 Hz, 1H, Ar-H), 7.93-7.88 (m, 2H, Ar-H), 7.70 (d, *J* = 8.4 Hz, 1H, Ar-H), 7.61-7.35 (m, 6H, Ar-H and alkene-H), 7.39 (d, *J* = 16.0 Hz, 1H, alkene-H), 6.90 (s, 1H, pyran-H). ^19^F NMR (400 MHz, DMSO-*d*_6_, ppm): *δ* -131.85. ^13^C NMR (400 MHz, DMSO-*d*_6_, ppm): *δ* 158.3, 153.9, 153.2, 152.4, 151.6, 137.2, 135.9, 126.6, 126.2, 125.1, 119.6, 119.4, 117.5, 117.5, 116.2, 112.3, 112.2, 112.1, 107.1, 60.8.

DCM-2F-OH (20.0 mg, 0.0574 mmol), 4-(bromomethyl)phenylboronic acid pinacol ester (26.6 mg, 0.0861 mmol), anhydrous sodium sulfate (20.4 mg, 0.1435 mmol), and cesium carbonate (93.5 mg, 0.287 mmol) were added to a 50 mL two-necked flask and dissolved in acetonitrile (15 mL). The mixture was stirred at room temperature for 24 h. After completion of the reaction, the insoluble materials were removed by filtration, and the filter cake was washed with dichloromethane. The combined filtrate was concentrated under reduced pressure and purified by column chromatography (DCM/MeOH, v/v, 50/1) to afford the product as a yellow solid (8.1 mg, yield 25.0%).^1^H NMR (400 MHz, DMSO-*d*_6_, ppm): *δ* 8.74 (d, *J* = 8.4 Hz, 1H, phenyl-H), 7.96 (t, *J* = 7.6 Hz, 1H, phenyl-H), 7.75 (d, *J* = 8.0 Hz, 1H, phenyl-H), 7.61-7.44 (m, 7H, phenyl-H), 6.99 (d, *J* = 8.8 Hz, 2H, phenyl-H), 6.83 (s, 1H, pyran-H), 5.16 (s, 2H, -OCH_2_-), 1.29 (s, 12H, Bpin). ^13^C NMR (400 MHz, DMSO-*d*_6_, ppm): *δ* 156.77, 155.53, 153.90, 152.26, 151.35, 138.78, 135.05, 133.93, 126.94, 125.71, 124.12, 120.72, 118.38, 116.47, 116.35, 115.07, 111.52, 111.39, 106.79, 83.13, 74.75, 60.45, 24.07. HRMS (ESI positive ion mode for [M+H]⁺): Calcd. for C_33_H_28_N_2_O_4_BF_2_^+^: 565.2105; found: 565.2108.

**2. Characterization of compounds**

**Figure S1.** ^1^H NMR spectrum of DCM-OH in DMSO-*d*_6_.

**Figure S2.** ^13^C NMR spectrum of DCM-OH in DMSO-*d*_6_.

**Figure S3.** ^1^H NMR spectrum of DCM-B in CDCl_3_.

**Figure S4.** ^13^C NMR spectrum of DCM-B in CDCl_3_.

**Figure S5.** HRMS spectrum of DCM-B.

**Figure S6.** ^1^H NMR spectrum of DCM-2F-OH in DMSO-*d*_6_.

**Figure S7.** ^13^C NMR spectrum of DCM-2F-OH in DMSO-*d*_6_.

**Figure S8.** ^19^F NMR spectrum of DCM-2F-OH in DMSO-*d*_6_.

**Figure S9.** ^1^H NMR spectrum of DCM-2F-B in DMSO-*d*_6_.

**Figure S10.** ^13^C NMR spectrum of DCM-2F-B in DMSO-*d*_6_.

**Figure S11.** HRMS spectrum of DCM-2F-B.

**4.** **Representative H₂O₂-responsive probes reported in recent years**

**Table S1.** Comparison of DCM-2F-B with representative H₂O₂-responsive fluorescent probes.

| Probe | λ_em_ (nm) | LOD (μM) | Detection mode | Sample types | Ref. |
| --- | --- | --- | --- | --- | --- |
| DCM-2F-B | 678 | 0.03 | Turn-on | Cells/Tissue | This work |
| Mito-HT | 562 | 0.33 | Ratiometric | Mitochondria imaging | [S1] |
| TFP | 470 | 0.068 | FLIM | Neurons/Zebrafish | [S2] |
| TCS | 680 | 1.81 | Dual-channel | Cells/Zebrafish/Tissue | [S3] |
| DXM-CHO-B | 670 | 0.039 | Turn-on | Cells/Zebrafish | [S4] |
| HH | 598 | 0.0186 | Dual-channel | Cells/Zebrafish/Mice | [S5] |
| H₂O₂-RMP | / | 0.56 | MRI (T₂-weighted) | Mice | [S6] |
| HOP | 680 | 0.41 | Turn-on | Cells/Mice | [S7] |
| Mn@Zr-MOF/OPD | 646 | 0.04 | Ratiometric | Cells / Smartphone test strips | [S8] |

**5.** **pH-Dependent Fluorescence of DCM-2Cl-OH**

**Figure S12.** pH-dependent fluorescence intensity profile of DCM-2Cl-OH (10 μM) at various pH values (3–12).

**6.** **DFT Calculations for Boronate Ester Derivatives**

**Figure S13.** DFT-calculated frontier molecular orbital energy levels of the boronate ester-masked probes DCM-B, DCM-2F-B, and DCM-2Cl-B.

**Figure S14.** DFT-calculated molecular electrostatic potential (MEP) maps of DCM-2Cl-OH and DCM-2Cl-B. Red: electron-rich (negative potential); Blue: electron-poor (positive potential).

**7. Biocompatibility of compounds**

**Figure S15.** Cytotoxicity of DCM derivatives. Cell viability at each concentration represents mean ± SD from five replicate wells (*n* = 5).

**8. ROS Imaging Using a 30 min Pre-Incubation Protocol**

**Figure S16.** Cellular ROS imaging with DCM-2F-B using a 30 min probe pre-incubation protocol. RAW264.7 macrophages were incubated with DCM-2F-B (10 μM) for 30 min, followed by various treatments for 60 min. (A) Schematic illustration of the fluorescence response mechanism. (B) CLSM images of cells treated with H₂O₂. (C) Quantitative analysis of fluorescence intensity for the H₂O₂ treated group. (D) CLSM images of cells treated with PMA. (E) Quantitative analysis of fluorescence intensity for the PMA treated group. (F) CLSM images of cells treated with PMA plus PBS, apocynin, or NAC. (G) Quantitative analysis of fluorescence intensity for the groups shown in (F). Scale bar: 50 μm. The data are expressed as the mean ± SD (*n* = 3). Statistical significance was determined using one-way ANOVA for multiple group comparisons. *****p* < 0.0001.

**9. Experimental Section**

**Reagents and Instruments**

Dicyanomethylene-4H-pyran (DCM), 3,5-difluoro-4-hydroxybenzaldehyde, 3,5-dichloro-4-hydroxybenzaldehyde, 4-hydroxybenzaldehyde, 4-(bromomethyl)phenylboronic acid pinacol ester, piperidine, N-acetyl-L-cysteine (NAC), and hydrogen peroxide were purchased from Adamas. Cesium carbonate, anhydrous sodium sulfate, and lipopolysaccharide (LPS) were purchased from Macklin and Beyotime. All chemicals were of analytical grade and used without further purification. NMR spectra were recorded on a Bruker Avance III 400 MHz spectrometer. High-resolution mass spectra (HRMS) were obtained on a Waters LCT Premier XE mass spectrometer. UV-Vis absorption spectra were measured on an Agilent Cary 60 spectrophotometer. Fluorescence spectra were recorded on a Varian Cary Eclipse spectrofluorometer. Confocal laser scanning microscopy (CLSM) images were acquired on a Leica TCS SP8 microscope. Absorbance for MTT assays was measured on a Bio-Rad iMark microplate reader.

**Determination of Spectroscopic Properties**

Stock solutions of DCM-OH, DCM-B, DCM-2F-OH, and DCM-2F-B (1 mM) were prepared in DMSO and stored at -20 °C in the dark. Working solutions (10 μM) were freshly diluted in PBS/DMSO (7:3, v/v, pH 7.4, 100 mM) before each measurement. Absorption spectra were recorded from 300 to 800 nm. Fluorescence spectra were measured using excitation wavelengths corresponding to the maximum absorption of each compound.

**DFT Calculations**

Density functional theory (DFT) calculations were performed using the Gaussian 16 package at the B3LYP/6-31G(d,p) level. The ground state geometries of DCM-OH, DCM-2F-OH, DCM-2Cl-OH, and their boronate ester derivatives were optimized. Frontier molecular orbital energy levels and molecular electrostatic potential distributions were calculated to elucidate the electronic effects of halogen substitution.

**ROS Response Assay**

DCM-2F-B or DCM-B (10 μM) in PBS/DMSO (7:3, v/v, pH 7.4) was incubated with various concentrations of H₂O₂ (0-300 μM) at 37 °C for 60 min. Absorption and fluorescence spectra were then recorded. For kinetic measurements, the fluorescence intensity at 678 nm was monitored over 60 min. The detection limit was calculated from the linear calibration curve using the 3*σ*/slope method.

**ROS Selectivity Assay**

To evaluate the selectivity of DCM-2F-B toward H₂O₂, competitive experiments were performed against a panel of biologically relevant interfering species, including reactive oxygen species (O₂⁻, ONOO⁻, ClO⁻, ·OH), inorganic salts, enzymes, amino acids, and biomolecules. All reactive oxygen species were freshly prepared immediately before use following established protocols.

The following interfering substances were tested at the indicated concentrations: inorganic salts (Na₂CO₃, KCl, Na₂SO₄, CH₃COOK, NaCl, CaCl₂, NH₄Cl, K₂CO₃; 300 μM), enzymes (acetylcholinesterase, esterase, tyrosinase, alkaline phosphatase, horseradish peroxidase, monoamine oxidase A; 100 U/mL), biomolecules (human serum albumin, hyaluronic acid, A*β* protein; 100 μg/mL; L-glutamic acid and glycine; 100 μM), and deionized water was used as the blank control.

Superoxide anion (O₂⁻, 300 μM): A 10 mM stock solution was prepared by dissolving KO₂ (2.84 mg, 40 μmol) in 4 mL of anhydrous DMSO with the aid of ultrasonication for 10 min. The supernatant was collected after centrifugation and used immediately.

Peroxynitrite (ONOO⁻, 100 μM): Equal volumes of NaNO₂ (0.6 M) and HCl (0.6 M) were mixed in an ice bath, followed by rapid addition of an equal volume of H₂O₂ (0.7 M). After stirring for 1 min, an equal volume of NaOH (1.5 M) was added to quench the reaction. The resulting solution was treated with MnO₂ powder to decompose residual H₂O₂ and then filtered to obtain the ONOO⁻ stock solution. The concentration was calibrated by measuring the absorbance at 302 nm (ε = 1670 M⁻¹cm⁻¹), and the solution was diluted to 10 mM and stored at −20 °C.

Hypochlorite (ClO⁻, 300 μM): A 10 mM stock solution was prepared by diluting commercial NaClO solution with PBS.

**Hydroxyl radical-generating system (·OH, Fe²⁺/H₂O₂):** A 10 mM Fe²⁺ stock solution was prepared by dissolving anhydrous FeCl₂ (6.34 mg) in 5 mL of deionized water. A 10 mM H₂O₂ stock solution was prepared by diluting 30% H₂O₂ with PBS. Hydroxyl radicals were generated in situ through the Fenton reaction by sequentially adding Fe²⁺ and H₂O₂ stock solutions to the reaction mixture, with Fe²⁺ added first followed by H₂O₂. The final concentrations of Fe²⁺ and H₂O₂ were both 150 μM.

General procedure: To a microcentrifuge tube containing PBS/DMSO (7:3, v/v, pH 7.4, 100 mM) was added 10 μL of DCM-2F-B stock solution (1 mM in DMSO, final concentration 10 μM). An appropriate volume of freshly prepared interfering solution was then introduced to initiate the reaction, and the mixture was transferred to a 1 mL quartz cuvette for measurement. The mixture was incubated in a water bath at 37 °C for 1 h in the dark. Fluorescence spectra were recorded with *λ*_ex_ = 505 nm and *λ*_em_ = 678 nm. All experiments were performed in triplicate.

**Cell Culture and Biocompatibility**

RAW264.7 macrophages were cultured in DMEM supplemented with 10% fetal bovine serum and 1% penicillin-streptomycin at 37 °C in a humidified atmosphere containing 5% CO₂. Cytotoxicity was evaluated using the MTT assay. Cells were seeded into 96-well plates at a density of 1 × 10⁴ cells per well and cultured for 12 h. Various concentrations (0-20 μM) of DCM-OH, DCM-2F-OH, DCM-B, or DCM-2F-B were added and incubated for 24 h. MTT solution (20 μL, 5 mg/mL) was then added to each well and incubated for 4 h. The formazan crystals were dissolved in DMSO (150 μL), and the absorbance at 490 nm was measured. Cell viability was calculated relative to untreated control wells.

**Cellular Uptake**

RAW264.7 cells were seeded into confocal dishes (1 × 10⁵ cells per dish) and cultured for 12 h. The cells were then incubated with DCM-2F-B or DCM-2F-OH (10 μM) for 0.5, 1, 2, or 4 h. After washing three times with PBS, fluorescence images were acquired using CLSM (*λ*_ex_ = 550 nm, emission collected from 650 to 750 nm). Mean fluorescence intensities were quantified using ImageJ software.

**Detection of Exogenous H_2_O_2_ in Cells**

RAW264.7 cells were pre-incubated with DCM-2F-B (10 μM) for 1 h, followed by stimulation with various concentrations of H_2_O_2_ (0, 50, 100, 200, 400 μM) for an additional 1 h. After washing three times with PBS, CLSM images were acquired (*λ*_ex_ = 550 nm, 650–750 nm). Fluorescence intensities were quantified using ImageJ software.

**Detection of Endogenous ROS in Cells**

To induce inflammation, RAW264.7 cells were treated with LPS (1 μg/mL) for 24 h, followed by incubation with DCM-2F-B (10 μM) for 1 h. For inhibition experiments, cells were pre-incubated with NAC (20 mM) for 1 h before LPS stimulation and probe incubation. CLSM images were acquired under the same conditions. Fluorescence intensities were quantified and normalized to control groups.

**Animal Model and Tissue Staining**

Following the established protocols from previous AS studies^[S9-S12]^, ApoE⁻/⁻ mice were fed a high-fat diet for 8 weeks to establish an atherosclerosis model. Aortic valve tissues were dissected, embedded in OCT, and frozen sectioned. Tissue sections were subjected to H&E staining, Masson staining, *α*-SMA immunofluorescence, Oil Red O staining, DHE staining, and TNF-*α*/CD68 immunofluorescence according to standard protocols. For ROS imaging, tissue sections were incubated with DCM-2F-B (10 μM) for 1 h at 37 °C. For NAC inhibition, sections were pre-incubated with NAC (5 mM in PBS) for 1 h before probe incubation. CLSM images were acquired (λex = 550 nm, 650–750 nm) and fluorescence intensities were quantified using ImageJ.

**Statistical Analysis**

All data are presented as mean ± standard deviation (SD) from at least three independent experiments. Statistical comparisons between two groups were performed using Student's t test, and among multiple groups using one way ANOVA. Significance levels are indicated as **p* < 0.05, ***p* < 0.01, ****p* < 0.001, *****p* < 0.0001. n.s. denotes no significant difference.

**References**

[S1] C. Wang, Y. Wang, G. Wang, C. Huang and N. Jia, *Anal. Chim. Acta*, **2020**, *1097*, 230.

[S2] Z. Wu, M. Liu, Z. Liu and Y. Tian, *J. Am. Chem. Soc.*, **2020**, *142*, 7532–7541.

[S3] H. Jin, Z. Xu, H. Tang, W. Tang, T. Ma, T. Yu, H. Zhou, Y. Deng, Y. Zhang, H. Li and P. Yin, *Anal. Chem.*, **2026**, *98*, 12542.

[S4] Z. Zhou, C. Fang, J. Xie, Y. Zhang and H. Li, *Anal. Chem.*, **2026**, *98*, 13450.

[S5] Y. Gan, Y. Xu, T. Yu, Y. Li, X. Jiang, H. Jin, H. Li, Y. Zhang, P. Yin and J. S. Kim, *Adv. Sci.*, **2026**, DOI: 10.1002/advs.2026076679.

[S6] X. Fan, Y. Sun, J. Fu, H. Cao, S. Liao, C. Zhang, S. Huan and G. Song, *Biomaterials*, **2025**, *321*, 123292.

[S7] Y. Liu, J. Yuan, T. Zhang, X. Cai, M. Xu, X. Wang, R. Wang, B. Zhang, H.-L. Zhu and Y. Qian, *Adv. Sci.*, **2025**, *12*, e02360.

[S8] X. Chai, R. Zhao, M. Asif, A. Aziz, C. Dong, Y. Tang, S. Shuang and Y. Guo, *Chem. Eng. J.*, **2026**, *527*, 171828.

[S9] I. Ilyas, P. J. Little, Z. Liu, Y. Xu, D. Kamato, B. C. Berk, J. Weng and S. Xu, *Trends Pharmacol. Sci.*, **2022**, *43*, 920.

[S10] D. J. Tyrrell, K. M. Wragg, J. Chen, H. Wang, J. Song, M. G. Blin, C. Bolding, D. Vardaman III, K. Giles, H. Tidwell, M. A. Ali, A. Janappareddi, S. C. Wood and D. R. Goldstein, *Nat. Aging*, **2023**, *3*, 1576.

[S11] J. M. Johnston, S. E. Francis and E. Kiss-Toth, *Cardiovasc. Res*., **2018**, *114*, 1845.

[S12] M. C. Martinez-Campanario, M. Cortés, A. Moreno-Lanceta, L. Han, C. Ninfali, V. Domínguez, M. J. Andrés-Manzano, M. Farràs, A. Esteve-Codina, C. Enrich, F. J. Díaz-Crespo, B. Pintado, J. C. Escolà-Gil, P. García de Frutos, V. Andrés, P. Melgar-Lesmes and A. Postigo, *Nat. Commun.*, **2023**, *14*, 8316.
